# Intestinal Spirochetosis mimicking inflammatory bowel disease in children

**DOI:** 10.1186/1471-2431-12-163

**Published:** 2012-10-16

**Authors:** Rossana Helbling, Maria-Chiara Osterheld, Bernard Vaudaux, Katia Jaton, Andreas Nydegger

**Affiliations:** 1 Pediatric Gastroenterology Unit, Department of Pediatrics, University of Lausanne, Centre Hospitalier Universitaire Vaudois, Rue du Bugnon 46, CH-1011 Lausanne, Switzerland; 2 Institute of Pathology, University of Lausanne, Centre Hospitalier Universitaire Vaudois, Rue du Bugnon 48, CH-1011, Lausanne, Switzerland; 3 Pediatric Infectious Disease Unit, Department of Pediatrics, University of Lausanne, Centre Hospitalier Universitaire Vaudois, Rue du Bugnon 46, CH-1011, Lausanne, Switzerland; 4 Institute of Microbiology, University of Lausanne, Centre Hospitalier Universitaire Vaudois, Rue du Bugnon 48, CH-1011, Lausanne, Switzerland

**Keywords:** Intestinal spirochetosis, Brachyspira aalborgi, Brachyspira pilosicoli, Inflammatory bowel disease

## Abstract

**Background:**

Intestinal spirochetosis is an unusual infection in children and its clinical significance in humans is uncertain. The presence of these microorganisms in humans is well-known since the late 1800’s and was first described in 1967 by Harland and Lee by electron microscopy.

**Case presentation:**

This article reports the findings of one pediatric case, review of the current literature, and an overview of therapeutic options.

**Conclusion:**

A high degree of suspicion is required in cases presenting with abdominal pain, chronic diarrhoea and/or hematochezia associated with a normal endoscopic examination, thus emphasizing the importance of multiple biopsies throughout the colon.

## Background

Spirochetes are well-known pathogens in veterinary medicine. They are associated with diarrheal illness, malnutrition and failure to thrive in a wide range of animals (swine, poultry, dogs, cats, opossum, non-human primates and guinea pigs) causing important economic losses [1].

The presence of intestinal spirochetes in human faeces has been recognized in the late 1800’s [2]. The term *intestinal spirochetosis* was coined in 1967 by Harland and Lee who recognized spirochetes adherent to the cellular membrane of apical cells in the colonic epithelium [3]. This histological appearance is considered to be the hallmark of this infection.

The clinical significance of intestinal spirochetes in humans remains highly debated. Indeed, it is still unclear whether the finding of microorganisms coating the intestinal mucosal membrane is simply the reflection of a colonization process or the histological aspect of a disease [4].

## Case presentation

A 13 year-old boy presented to our paediatric gastroenterology outpatient clinic with a one month history of blood-stained diarrhoea, associated with urgency, weight loss of 1.5kg and asthenia. He had no complaint of abdominal pain, fever or anorexia. The onset of symptoms coincided with the end of a 4-day history of acute gastroenteritis.

**Figure 1:**
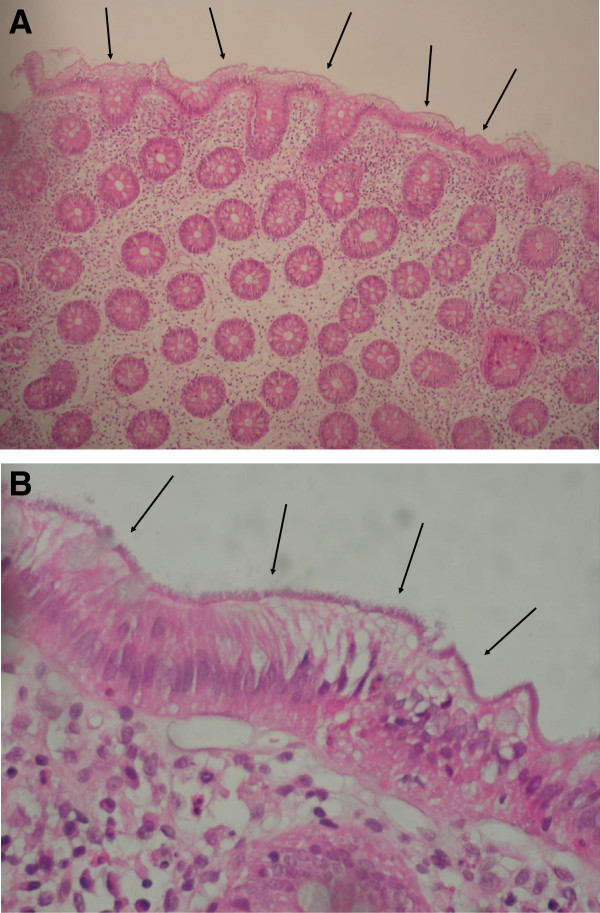
**A: Hematoxylin and eosin stain of colon biopsy specimen showing multitudes of organisms along apical border of enterocytes, resembling a “false brush border” (H&E, orig. mag. x10).B: Colonic mucosa with thick hematoxyphilic fringe on the brush border of the luminal surface (H&E, orig. mag. X40).**

**Figure 2:**
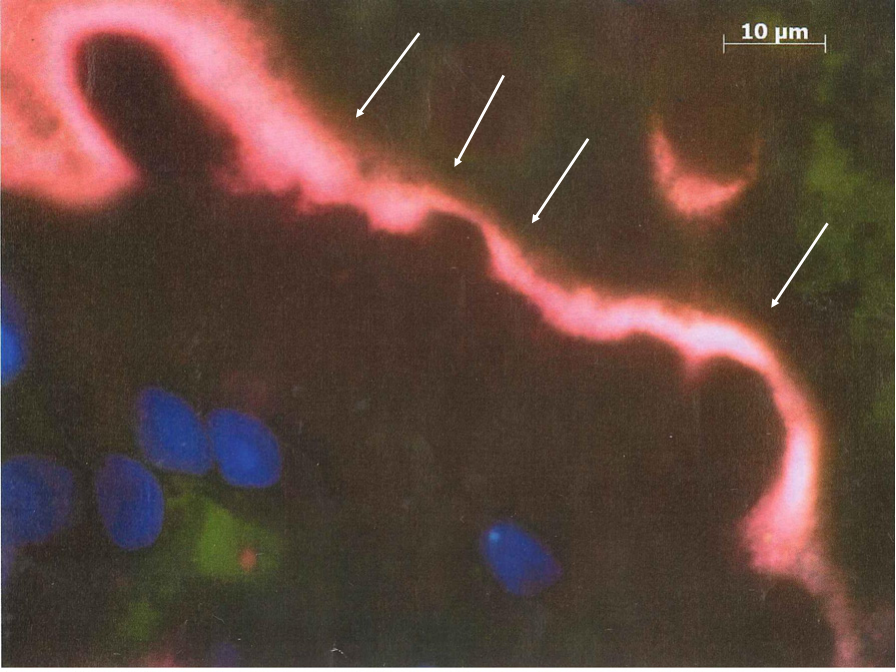
**Visualisation and identification of Brachyspira spp. in a colon biopsy specimen by FISH (white arrows).** Blue dots stand for counterstained cell nuclei.

His past medical history revealed a recurrent aphthous stomatitis, and his family history was positive for celiac disease (one maternal cousin), irritable bowel syndrome (one paternal uncle) and bowel cancer (paternal grandfather). His growth parameters were normal and the physical examination revealed palpable stool masses in the left lower abdomen. Based on the clinical presentation, the differential diagnosis included inflammatory bowel disease, celiac disease, infectious colitis and intestinal polyp. Laboratory investigations showed no biological markers for inflammation (normal full blood count, ESR: 5mm/h and CRP: 0.28mg/l) or celiac disease. Fecal calprotectin was not measured. Stool cultures for conventional enteric pathogens (*Salmonella, Shigella Campylobacter*) were negative. Upper and lower endoscopy revealed mucosal oedema in the sigmoid and rectum, which was probably due to the required bowel preparation for colonoscopy. Histological examination on conventional hematoxylin and eosin stain of the colonic biopsy specimens revealed a fuzzy band on the surface of the enterocytes. This finding was highly suggestive of intestinal spirochetosis (Figure 1). Interestingly, no specific inflammatory reaction or lesions were observed. The diagnosis of intestinal spirochetosis was confirmed by Fluorescence in situ hybridization (FISH), confirming the presence of *Brachyspira aalborgi* (Figure 2).

On the assumption that most intestinal spirochetes (obtained from veterinary specimens) are susceptible to amoxicillin, the patient was initially treated with this antibiotic (30mg/kg/day, divided into 3 doses) during 14 days, with subsequent cessation of rectal bleeding but persistent mucous diarrhoea. Additional treatment with metronidazole (30mg/kg/day, divided into 3 doses) for 10 days resulted in complete resolution of symptoms, therefore, no follow-up endoscopy was performed.

## Methods

A Medline search was performed for all studies published from 1993 to December 2011 using the Medical Subject Heading and keywords: “intestinal spirochetosis” and “all child (0–18 years)”. Articles in any other language than English have been excluded. All studies retrieved were considered and data from relevant’ ones were presented in Table 1 and discussed in the text.

**Table 1:** Description of 25 cases of pediatric intestinal spirochetosis (IS)

**Ref.**	**Age (y)**	**Sex**	**Signs and Symptoms**	**Endoscopic findings**	**Histologic findings**	**Treatment**	**Comments**
[5]	6	F	Rectal bleeding, abdominal pain, pruritus ani	Normal	IS and mild inflammation	Piperazine	Improvement
	2	M	Intermittent bloody diarrhoea	Distal colitis	IS and mild to moderate infiltration with inflammatory cells	Mebendazole (Ascaris in stool)	Improvement
	13	M	Recurrent fever, abdominal pain, joint pain, mouth and penile ulcers	Normal	IS and mild patchy inflammation	No treatment	No follow-up information
[6]	7.5	M	Periumbilical pain, frequent bloody stools, urgency, poor appetite and weight loss	Mild rectal granularity	IS	1) Metronidazole	Persistence of diarrhea; eradication on endoscopicfollow-up at 6 months
						2) Neomycine	
	8	F	Acute abdominal pain, vomiting and fever	Not done	IS in resected appendix	No treatment	Improvement
[7]	12	M	Intermittent vomiting, diarrhoea, weight loss, headaches and fatigue	Normal	IS and mild focal colitis	Metronidazole and Amoxicillin (7 days)	Improvement
	12	M	Recurrent abdominal pain	Normal	IS and mild focal cryptitis in the caecum	1) Penicillin V and Metronidazole (7 days)	Persistence of symptoms and IS; after second course of Metronidazole eradication and improvement
						2) Metrodidazole (800mg 3x/d for 7 days)	
	16	F	Recurrent colicky right upper quadrant pain	Normal	IS	Metronidazole and Amoxicillin (10 days)	Improvement
	9	F	Intermittent diarrhoea and rectal bleeding	Normal	IS	Metronidazole and Amoxicillin (10 days)	Improvement
[8]	5	F	Abdominal pain, diarrhoea and rectal bleeding	Rectal oedema and enterobiasis	IS	Erythromycin (40mg/kg/d for 10 days)	Rectal bleeding ceased but abdominal pain recurred, no follow-up
	7	M	Abdominal pain and diarrhoea	Slight proctitis	IS	Doxycycline (200mg day1, then 100mg/d for 8 days )	Persistence of abdominal pain despite endoscopic eradication
	4	F	Mucus and bloody stools	Proctitis, juvenile polyp	IS	Clarithromycin (50mg/kg/d for 10 days)	Improvement
	10	F	Diarrhoea and rectal bleeding	Rectal hyperaemic membranes	IS	Clarithromycin	Improvement
	13	M	Abdominal pain, nausea and weight loss	Slight inflammation of rectum	IS and HP- positive gastritis	1) Clarithromycin and Amoxicillin	Persistence of IS, eradication after Clarithromycin and Metronidazole
						2) Clarithromycin and Metronidazole (500mg 2x/d and 400mg 3x/d 7 days)	
	8	M	Recurrent abdominal pain	Juvenile polyp	IS and low-grade chronic inflammation	1) Penicillin V	Improvement after Erythromycin
						2) Erythromycin (40-50mg/kg/d for 10 days)	
	15	F	Abdominal pain and rectal bleeding	Normal	IS	Clarithromycin (500mg 2x/d for 14 days)	Improvement but persistence of rectal bleeding; absence of IS on follow-up rectoscopy
	14	F	Abdominal pain	Normal	IS	Metronidazole (400mg 3x/d for 10 days)	No improvement; absence of IS on follow-up colonoscopy
[9]	9	M	Recurrent abdominal pain, diarrhea and rectal bleeding	Mild erythema of rectal mucosa	IS and hypereosinophilia	Erythromycin (40mg/kg/d for 10 days)	Improvement
[10]	9	M	Rectal bleeding, diarrhea, tenesmus, flatulence and weight loss	Normal	IS and mild epithelial reactive changes	No treatment	Improvement
[11]	4	F	Abdominal discomfort, rectal bleeding and weight loss	Mucosal erosions, hyperemia, 2 juvenile polyps	IS and inflammatory infiltrate	Metronidazole	Improvement
[12]	11	F	Intermittent abdominal pain and rectal bleeding	Normal	IS	1)Metronidazole (250mg 3x/d)	No improvement, additional course of Metronidazole and Vancomycin with persistence of IS, no follow-up information
						2) Metronidazole (1000mg/d for 2 months and 750 mg/d for 14 days)	
						3) Vancomycin (7 days)	
	6	M	Stomach cramps, rectal bleeding, intermittent diarrhea, rectal prolapse	Normal	IS	Metronidazole (250mg 2x/d)	Improvement, but alternating constipation with watery diarrhea and rectal prolapse
	17	F	Recurrent abdominal pain, nausea, vomiting	Normal	IS and mild eosinophilic inflammatory infiltrate	No treatment	No follow-up information
	11	F	Right lower quadrant pain	Not done	Appendicitis and IS in resected appendix	Cefoxitin (30mg/kg/dose, 4 doses)	No follow-up information
	10	M	Periumbilical and epigastric pain, nausea, fever	Not done	Appendicitis and IS in resected appendix	No treatment	No follow-up information

## Results

To the best of our knowledge, there were 8 articles describing a total of 25 paediatric cases of intestinal spirochetosis [5-12]. Three patients presented spirochetes on histopathological examination of a resected appendix without endoscopy. Sex distribution was similar (13 girls, 12 boys) with a median age of 9.5 years (range: 2–17 years). Patients mainly presented with abdominal pain, diarrhoea and rectal bleeding with, apart from aspecific signs of mild inflammation, normal endoscopic findings. Table 1 summarizes those 25 patients with respect to their age, symptoms, endoscopic and histological findings and treatment. Many patients had more than one treatment due to persistence of symptoms, although metronidazole seemed to be the favourite therapeutic agent. Two out of 5 patients improved without treatment and the overall outcome was favourable in 17 patients (68%).

## Discussion

Adhesion of spirochetes to the brush border mucosa resulting in its thickening as seen on light microscopy can be observed worldwide in both children and adults [1]. Diagnosis is usually made by histopathology.

Spirochetes are currently divided into three phylogenetic groups: *Spirochaetaceae* including *Borrelia, Spirochaeta, Spironema* and *Treponema; Leptospiraceae* including *Leptonema* and *Leptospira*; and finally *Brachyspiraceae* containing the intestinal spirochetes of *Brachyspira (Serpulina)*[1]. *Brachyspira aalborgi* (*B. aalborgi*) and *Brachyspira pilosicoli* (*B. pilosicoli*), the two members of *Brachyspiraceae* family, are both described in humans and are considered as cause of human intestinal spirochetosis [13]. The anaerobic weakly β-haemolytic intestinal spirochete *B. aalborgi*, was first isolated in 1982 in the stool of a patient from Denmark [14], whereas *B. pilosicoli* is responsible for colitis and typhlytis in pigs, poultry and other species, acting more as zoonotic agent [15]. Since 1997, *Brachyspira spp*. have been included in the list of human enteropathogenic bacteria [16]. These two spirochetes are slow growing fastidious anaerobes and require specific media, with estimated growth times of 6 days for *B. pilosicoli* and up to 2 weeks for *B.aalborgi*[17]. Morphologically, they are coiled Gram-negative bacilli and are mobile in liquid environment due to the presence of flagella. Generally they are considered non invasive microorganisms but systemic spread of *B.pilosicoli* has been documented by culture of blood specimens obtained from critically ill patients [18]. Concomitant infection by *B.aalborgi* and *B.pilosicoli* is described but rare [19]. These spirochetes are susceptible to different antibiotics, such as metronidazole, meropenem, chloramphenicol, ceftriaxone and tetracycline, whereas a 60% resistance rate to ciprofloxacine has been observed [20]. Little is known about the way of transmission, but it seems likely to occur by faecal-oral route (contaminated water, colonized/infected faeces) [19]. Due to the higher prevalence in homosexual men, sexual transmission has been suggested as well [21]. Possible co-infection with others microorganisms like *Helicobacter pylori*, *Enterobius vermicularis*, *Shigella flexneri*, *Neisseria gonorrhoeae*, *Entamoeba histolytica*, *Blastocystis hominis*, and *Ascaris* is possible, rendering clinical significance difficult [2].

The prevalence varies considerably in geography and immune condition. In developed countries for example ranges between 1.1-5% can be observed, with an increase in homosexual men and HIV positive patients [1]. In a recent study from Japan the incidence of human intestinal spirochetosis in patients aged from 35 to 75 years was 0.4% and therefore lower than in Western countries [22]. The paucity of epidemiologic data is probably due to several reasons: firstly: the endoscopist not always takes routine multiple biopsies in healthy looking mucosa; secondly: the pathologist actively has to look for spirochetes and can easily miss them if not familiar with its appearance and thirdly: it is not a routine diagnosis by microbiologist either [11].

In most cases, intestinal spirochetosis is asymptomatic and presents as accidental findings during a screening colonoscopy for other reasons [23]. However, infected children usually complain of persistent diarrhoea, rectal bleeding, constipation, abdominal pain, weight loss, failure to thrive, nausea and lack of appetite [24]. The severity of disease can vary from asymptomatic colonisation to invasive and rapidly fatal progression [25], but there appears to be no correlation with degree of immunodeficiency in HIV positive patient and the extent of disease [1]. Due to this unspecific presentation, differential diagnosis should include inflammatory bowel disease, infectious, ischemic or pseudomembranous colitis and rectocolic carcinoma [24]. Colonic involvement is documented from distal to proximal, including rectum and appendix [26]. Mucosal appearance on endoscopy is not helpful in making the diagnosis, as it can be normal, polypoid, and erythematous, or just show unspecific lesions [27]. In a large Australian case series of 113 adult patients presenting with intestinal spirochetosis, 90 percent of colorectal specimens showed no morphological alterations, whereas the remaining cases had other possible causes for inflammation [23]. Histological appearance of a diffuse blue-fringe (better seen in hematoxylin-eosin or a silver stain), 3-6μm thick, along the border of intestinal epithelial layer, referred to as the « false brush border » is highly suggestive [1]. The surrounding cytostructure may show inflammation with slight oedema, infiltrate of monocytes, lymphocytes, plasma cells and neutrophils in the lamina propria, as well as elongated and hyperplastic crypts [28]. On electron microscopy, spirochetes are attached perpendicularly to the epithelial membrane of the enterocytes and the microvilli appear shortened or depleted [3]. Analysis of specimens from infected individuals even though rare has shown spirochetal invasion of colonic epithelial cells, macrophages, goblet cells and Schwann cells [29]. Histologically it is not possible to distinguish *B.pilosicoli* from *B.aalborgi*, therefore genetic methods have been developed in order to identify *Brachyspira* species from stool or tissue samples. Fluorescent in situ hybridization using oligonucleotide probes targeting 16S or 23S rRNA of *B. aalborgi* and *B. pilosicoli* allow visualisation and identification of this microorganism [28].

Treatment strategies have been proposed for intestinal spirochetosis eradication, including macrolids and clindamycin, but metronidazole seems to be the drug of choice, with a dose regimen of 500mg 3 times a day for 10 days in adults and 15mg per kg bodyweight 3 times per day for 5 days in children [19]. At the moment, there is lack of evidence regarding the most effective antibiotic agent as treatment response is variable and sometimes even ineffective supporting the hypothesis that these microorganisms are harmless commensals in humans, rendering specific treatment questionable [1]. Spontaneous recovery has been described after a prolonged period for up to 8 months [8].

## Conclusions

Intestinal spirochetosis may be more frequent than suspected and clinicians should take into account this disease, especially in case of persistent diarrhoea without any other reason. Diagnosis can only be made by an experienced pathologist and microbiologist and requires a lower endoscopy with multiple biopsies throughout the colon.

## Competing interests

The authors declare that they have no competing interests.

## Authors’ contributions

RH drafted the manuscript and collected data related to the subject; BV was involved in revising critically the manuscript and helped to draft the manuscript. AN participated in the design of the manuscript and the coordination. All authors read and approved the final manuscript.

## Pre-publication history

The pre-publication history for this paper can be accessed here:

http://www.biomedcentral.com/1471-2431/12/163/prepub

## Author details

^1^Pediatric Gastroenterology Unit, Department of Pediatrics, University of Lausanne, Centre Hospitalier Universitaire Vaudois, Rue du Bugnon 46, CH-1011 Lausanne, Switzerland. ^2^Institute of Pathology, University of Lausanne, Centre Hospitalier Universitaire Vaudois, Rue du Bugnon 48, CH-1011 Lausanne, Switzerland. ^3^Pediatric Infectious Disease Unit, Department of Pediatrics, University of Lausanne, Centre Hospitalier Universitaire Vaudois, Rue du Bugnon 46, CH-1011 Lausanne, Switzerland.^4^Institute of Microbiology, University of Lausanne, Centre Hospitalier Universitaire Vaudois, Rue du Bugnon 48, CH-1011 Lausanne, Switzerland.
